# Multimodal measurements enhance insights into emotional responses to immediate feedback

**DOI:** 10.3389/fpsyg.2023.1294386

**Published:** 2024-02-01

**Authors:** Anne Horvers, Inge Molenaar, Heleen Van Der West, Tibor Bosse, Ard W. Lazonder

**Affiliations:** Behavioural Science Institute, Radboud University, Nijmegen, Netherlands

**Keywords:** emotional responses, immediate feedback, adaptive learning technologies, physiological arousal, multimodal measurements

## Abstract

Adaptive learning technologies often provide students with immediate feedback on task performance. This feedback can elicit various emotional responses, which, in turn, influence learning. Most recent studies capture these emotions by single data streams, contradicting the multi-componential nature of emotion. Therefore, this study investigated 32 university students solving mathematical problems using an adaptive learning technology. Students received immediate feedback on every step in the solution process, after which their physiological, experiential and behavioral responses to this feedback were recorded. Physiological arousal was measured by electrodermal activity, valence was measured by self-reports (experiential), and emotion types were measured by observations of facial expressions (behavioral). Results showed more peaks in electrodermal activity after feedback than was expected based on chance. These responses were comparable in strength after feedback on failure and success. Students’ experiential responses conveyed mostly positive valence after feedback on success and mostly negative valence after feedback on failure. Behavioral observations showed more negative than positive emotion types after feedback on failure and more positive than negative emotion types after feedback on success. These results show that physiological arousal is a valuable objective indicator of emotional responses after immediate feedback but should be accompanied by other data streams in order to understand students’ emotional responses. Both valence and emotion types can be used for this purpose. These outcomes pave the way for designing adaptive learning technologies that take students’ emotions into account.

## 1 Introduction

In recent years, computer-assisted learning, intelligent tutoring systems and adaptive learning technologies (ALTs) have increasingly become prevalent in education across the globe (Martin et al., 2020; Baker, 2021). These systems have been developed to promote individual students’ learning by providing automated feedback on their task performance (van Lehn, 2011; Aleven et al., 2016; Pardo et al., 2019). This feedback can be delivered in real-time during the learning process, which is known to increase learning effectiveness (Tärning, 2018; Deeva et al., 2021). Most contemporary learning technologies, such as ALTs, go beyond providing immediate feedback by adapting the difficulty of future practice problems to individual students’ current task performance. Students’ answers are utilized to infer their ability level, which the ALT’s underlying algorithm uses to determine the difficulty of the next practice problem (Elo, 1978; Klinkenberg et al., 2011). However, this algorithm is solely based on cognitive achievements and disregards students’ emotions (Aleven et al., 2016), which play a crucial role in the learning process and directly influence students’ learning (D’Mello, 2017; Pekrun, 2022). For example, negative emotions hamper learning (Götz and Hall, 2013; Loderer et al., 2020) and affect students’ effort, perception and use of learning strategies (Eteläpelto et al., 2018; Obergriesser and Stoeger, 2020). Moments of success and failure in learning can elicit a wide range of emotional responses, and the same goes for feedback on learning task performance (Peterson et al., 2015; Lipnevich et al., 2021). In other words, there is good reason to examine the possibility of ALTs taking emotions into account. A first step in that direction is to better understand which emotions are triggered by immediate feedback.

This study aimed to gain insight into students’ emotional responses to immediate feedback given by an ALT. Emotions are seen as multi-componential in nature in this study, consisting of the dimensions arousal and valence (Russell, 1980; Pekrun, 2006). This study extends previous research by analyzing multimodal data streams to capture these dimensions: measures of physiological arousal via electrodermal activity (EDA), self-reported valence (experiential) and observations of emotion types via facial expressions (behavioral). Additional innovative features include the instant measurement of emotional responses (rather than at the end of a learning session) to immediate feedback on every step in the solution process (as opposed to feedback on the final solution or delayed feedback). The next sections elaborate on the roles emotions can play during learning, measures of emotions, and the different types of emotional responses to feedback.

### 1.1 Emotions in learning

Learning and human emotions are reciprocally related: emotions affect learning directly, and success or failure during the learning process influences students’ emotions (Pekrun, 2006). Emotions also affect students’ effort, perception and use of learning strategies (Eteläpelto et al., 2018; Pekrun, 2022). Emotions can either enhance or impede learning. For example, when students feel frustrated, confused or bored, their learning is negatively impacted, while feelings of enjoyment or pride have a positive influence on students’ learning (Götz and Hall, 2013; Loderer et al., 2020). However, overcoming a state of confusion can also benefit learning, which illustrates that the interplay between emotion and learning can vary among students (Baker et al., 2010; Graesser, 2020). That is, some students may prefer easy tasks to avoid negative emotions due to failure, whereas others like to be challenged by difficult tasks and experience fewer emotions when they do not succeed (Baker et al., 2010).

These emotions students experience can be defined from a categorical and dimensional perspective. Categorical theories divide emotions into different types, such as fear, anger, happiness, surprise, disgust and sadness (Ekman, 1999). Each emotion type is associated with a distinct facial expression and action tendencies (Coppin and Sander, 2021). However, it has been argued that these basic emotion types bear little relationship with learning (Kort et al., 2001). Dimensional theories of emotion, by contrast, do have this connection and address the multi-componential nature of emotions by portraying emotions by the continuous components arousal and valence (Russell, 1980; Pekrun, 2006). Arousal indicates the amount of physiological activation of the body that occurs when an emotion is triggered, while valence refers to the pleasantness of an emotion, which can range from positive to negative (Russell, 1980; Pekrun, 2017).

The Control-Value Theory (CVT) integrates these perspectives, specifically focusing on emotions during learning (i.e., achievement emotions) and is widely used in educational research (Pekrun, 2017). This theory argues that achievement emotions can differ in object focus, with a distinction between activity emotions that occur during learning (e.g., boredom during a learning task) and outcome emotions related to success and failure in the past or future (e.g., anxiety related to future failure or pride related to past success) (Jarrell et al., 2017; Pekrun, 2017). Anxiety is, for instance, seen as an emotion with a negative valence, high activation and an outcome focus (Pekrun et al., 2007). The effects of especially positive deactivating and negative activating emotions on learning are complex (Pekrun and Linnenbrink-Garcia, 2014). Experiencing positive deactivating emotions (e.g., relaxation) can reduce students’ effort and negatively influence learning, contrary to positive activating emotions (e.g., enjoyment and pride) (Wu and Yu, 2022). Negative activating emotions (e.g., frustration and anxiety) are shown to impede learning, but can also enhance students’ effort to perform better (Graesser and D’Mello, 2012; Pekrun, 2017; Cloude et al., 2021; Taub et al., 2021). In this study, emotions are conceptualized as multi-componential using the CVT.

The multi-componential nature of emotions, as described by dimensional theories, points to differences in the expression and experience of these emotions between humans (Harley et al., 2015; Azevedo et al., 2022). This emotional experience is also influenced by a combination of a student’s appraisal of a learning situation and the associated emotional response, as students take into account their perceptions of control and evaluations of task value (Pekrun, 2017). Moreover, different psychological subsystems are at play when a student feels anxious, including affective (feeling nervous), cognitive (being worried), motivational (avoidance), expressive (nervous face), and physiological (high bodily activation) processes (Kleinginna and Kleinginna, 1981; Pekrun, 2006; Ortony et al., 2022). Considering emotions as a multi-componential construct and measuring it as such is recommended by recent studies as well (Li et al., 2021). Previous research typically recorded students’ physiological, experiential, and behavioral responses to personally meaningful stimuli (Mauss and Robinson, 2009; Horvers et al., 2021). These physiological responses involve the reaction of the body when an emotion is evoked (Dawson et al., 2016). Experiential responses refer to the subjective personal experience of an emotion, and behavioral responses concern the observable behavioral reactions (Mauss and Robinson, 2009). These responses provide the opportunity to measure emotions in a multi-componential way, this approach will be used in this study.

### 1.2 Emotional responses to feedback during learning

Feedback can cause various physiological, experiential and behavioral responses (Jarrell et al., 2017). Variations in EDA (physiological arousal) can occur when students receive feedback on their performance. For example, Aghaei Pour et al. (2010) found cross-student differences in an unspecified set of physiological features, while Malmberg et al. (2019a) showed that synchrony in the EDA’s above-threshold peaks occurred when students discussed collective feedback. Feedback can elicit valence (experiential) ranging from positive to negative and different facial expressions (behavioral) (Peterson et al., 2015; Lipnevich et al., 2021). Evidence regarding the relationship between emotional responses and feedback on success (FOS) and feedback on failure (FOF) is typically mixed, possibly resulting from differences in individual’s appraisals of a learning situation (Pekrun, 2017). Some studies concluded that FOF leads to negative emotions, such as frustration, and FOS leads to positive emotions (D’Mello et al., 2010; Lipnevich et al., 2021). Other studies showed that FOF elicits particularly intense and negative emotions (Rowe et al., 2014; Hill et al., 2021), which can linger longer than positive emotions and resurface with greater intensity on future tasks (Hill et al., 2021). These emotional responses can also impact students’ actions — that is, positive emotions can motivate students to try harder and improve by facilitating the evaluation of their learning (Pitt and Norton, 2017). When students get feedback that their answer is incorrect, they can become frustrated or anxious and, hence, discouraged to perform the next task (Vogl and Pekrun, 2016). However, these same negative emotions can also motivate some students to perform better on the upcoming task (Vogl and Pekrun, 2016; Lim et al., 2020). Repeated instances of FOF undermine students’ sense of control and result in negative emotions (Pekrun, 2006). Feedback that an answer is correct (i.e., FOS) can lead to feeling in control of learning, which can again lead to pride (Lipnevich et al., 2021).

This study adds to the existing body of research by (1) investigating immediate feedback on every step in the solution process, (2) measuring emotional responses during the learning process: continuously and after every instance of feedback, and (3) using a multimodal approach to capture these emotional responses. The above-mentioned insights from previous research and existing theories are mainly derived from studies investigating emotional responses to delayed feedback, either given by teachers when students struggle and ask for help or by technologies after the completion of a full learning session (Hill et al., 2021; Lipnevich et al., 2021). Even though ALTs and other technologies are particularly suitable for providing students with immediate feedback, most recent studies focused on detecting emotions during the learning process without attending to the role of feedback (Loderer et al., 2020; Lal et al., 2021). The studies that do focus on feedback investigated it after a task has been completed (Aghaei Pour et al., 2010; D’Mello et al., 2010), while contemporary technologies also provide opportunities to provide feedback on every step in the solution process (Molenaar, 2022). It remains unclear whether and to what extent these previous insights generalize to situations where students receive immediate feedback. The present study will adopt a fine-grained approach to immediate feedback to answer this question.

Secondly, extant research generally assessed emotional responses at the end of the learning session (Jarrell et al., 2017; Hill et al., 2021) or even after a week’s delay (Peterson et al., 2015; Lipnevich et al., 2021). These retrospective measures miss out on the rapidly changing nature of emotions which causes the dynamics of feedback and emotion to happen in less than a second (Pekrun, 2006). As ALTs provide immediate feedback, emotional responses to this feedback should be instantly assessed by fine-grained measures (Pekrun, 2006; D’Mello, 2013). Physiological arousal is a promising measure to instantly capture emotional responses because the intensity of and fluctuations in arousal can be measured in real-time (Jarrell et al., 2017; Malmberg et al., 2019a).

Finally, most previous research on the connection between feedback and emotions used unimodal approaches, for example, by capturing only experiential responses through semi-structured interviews (Lim et al., 2020; Hill et al., 2021) or self-report questionnaires (Jarrell et al., 2017; Lipnevich et al., 2021). However, considering emotions as a multi-componential construct is recommended by recent publications (Li et al., 2021). Using a single data stream has constraints, such as the focus on one aspect of emotion and the subjective nature of self-report data (Pekrun, 2020). In addition, physiological, experiential, and behavioral responses are weakly related, suggesting that each portrays a unique aspect of a person’s emotional responses (Mauss and Robinson, 2009), that cannot be measured by the other responses (Egger et al., 2019). For example, physiological arousal can be successfully measured with EDA, whereas valence cannot (Mauss et al., 2005; Mauss and Robinson, 2009; Horvers et al., 2021). As it remains unclear how students emotionally respond to feedback during learning, a multimodal approach seems more appropriate (Peixoto et al., 2015; Egger et al., 2019). By using physiological arousal as measured by EDA (physiological responses), self-reported valence (experiential responses) and emotion types as measured by observations of facial expressions (behavioral responses) after every feedback event, this study aimed to gain a detailed understanding of emotional responses to immediate feedback in ALTs, which paves the way for designing ALTs that take students’ emotions into account.

### 1.3 Research questions and hypotheses

Although ALTs provide students with immediate feedback in real time, hardly any research has been done on emotional responses to this feedback. However, gaining an understanding of these relationships is important for designing ALTs that can take emotions into account. This study, therefore, aimed to investigate emotional responses to feedback given by an ALT immediately after students entered a calculation into the system. Physiological, experiential and behavioral responses were measured in a multimodal approach. Physiological responses were captured by physiological arousal as measured continuously by EDA. Experiential responses were assessed through self-reported valence and behavioral responses by capturing emotion types as measured by observations of facial expressions, both after every instance of feedback. These data streams were analyzed to answer the following research questions: (1) To what extent does immediate feedback trigger peaks in students’ EDA (physiological arousal)? (2) Which experiential (valence) and behavioral responses (emotion type) of students are triggered by immediate feedback?

As previous research has shown that feedback can elicit emotions in students and physiological arousal can vary after feedback (D’Mello, 2017; Malmberg et al., 2019a), we expected that that feedback will generate above-threshold peaks (EDA) at an above-chance level (hypothesis 1). As feedback on failure (FOF) elicits particularly strong emotions (Rowe et al., 2014; Hill et al., 2021), students’ physiological responses were expected to be stronger. That is, there will be more above-threshold peaks in EDA, a higher amplitude sum and higher mean phasic activity within response window for FOF than for feedback on success (FOS) (hypothesis 2). As most previous research showed that FOF would predominantly elicit negative emotions while FOS would mainly elicit positive emotions (D’Mello et al., 2010; Lipnevich et al., 2021), the third hypothesis predicted that students’ experiential responses would indicate predominantly negative valence after FOF and positive valence after FOS. A similar pattern was expected to occur for behavioral responses, meaning that students will exhibit predominantly negative emotion types after FOF and positive emotion types after FOS (hypothesis 4) (D’Mello et al., 2010; Lipnevich et al., 2021).

## 2 Materials and methods

### 2.1 Participants

Data was gathered from 36 Dutch university students, but 4 of them had to be excluded from the sample due to technical problems during data collection. The remaining 32 participants were 24 women (75%) and 8 men (25%) aged 18–28 (*M* = 21.28, *SD* = 2.67). They studied at the faculty of arts (25%), faculty of medical sciences (15%), faculty of management (15%), faculty of law (5%), faculty of science (15%), and faculty of social sciences (25%). Thirteen participants were first-year bachelor students (40.6%), 4 were second-year bachelor students (12.5%), 5 were third-year bachelor (15.6%), 5 were fourth-year bachelor (15.6%), and 5 were master students (15.6%). Students signed active consent for participation in this study. The research has been independently reviewed by the Ethics Committee Social Sciences (ECSS) of the Radboud University, and there is no formal objection [ECSW-2020-14].

### 2.2 Design and procedure

**Figure 1:**
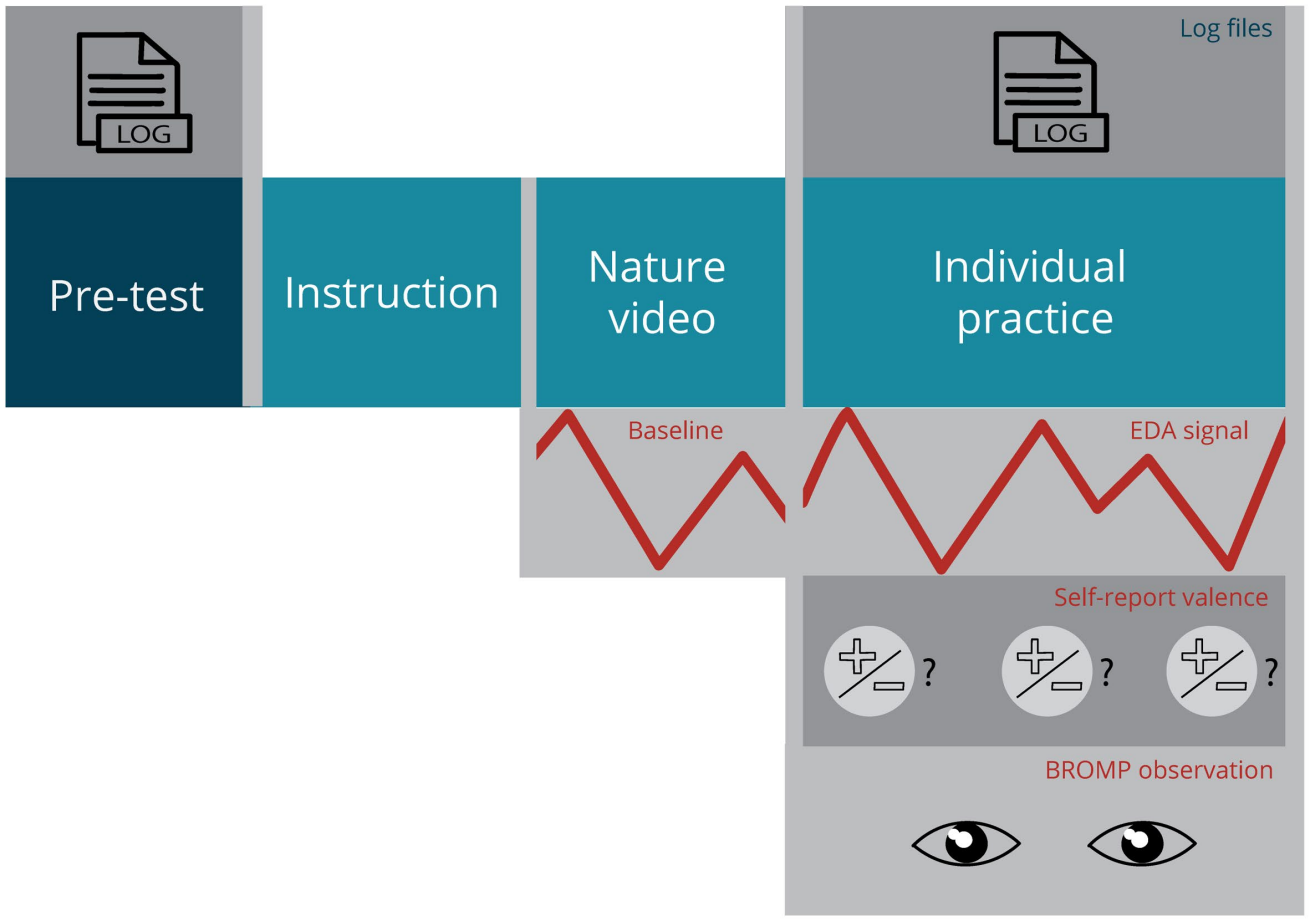
Study design.

This descriptive study administered a pre-test-only design (Figure 1) and took place in a research laboratory at the students’ university. During the main phase of the study, students worked with an ALT to solve mathematical problems using the quadratic formula. Prior to that, they took a pre-test that assessed their prerequisite math knowledge and received video instructions on the quadratic formula. Next, they watched a nature video to establish the EDA baseline. Students then solved three mathematics problems and received immediate feedback on every calculation they entered into the ALT. After every feedback event, students were prompted to report their experiential responses by indicating the valence of their emotional state on a 5-point scale. Their physiological arousal was measured through sweat gland activity using an EDA wristband during the entire session (see section 2.4.4). Behavioral responses were captured by observations of facial expressions indicating emotion types and were done after the session using video recordings of the participants’ faces.

### 2.3 Materials

#### 2.3.1 Adaptive learning technology

**Figure 2:**
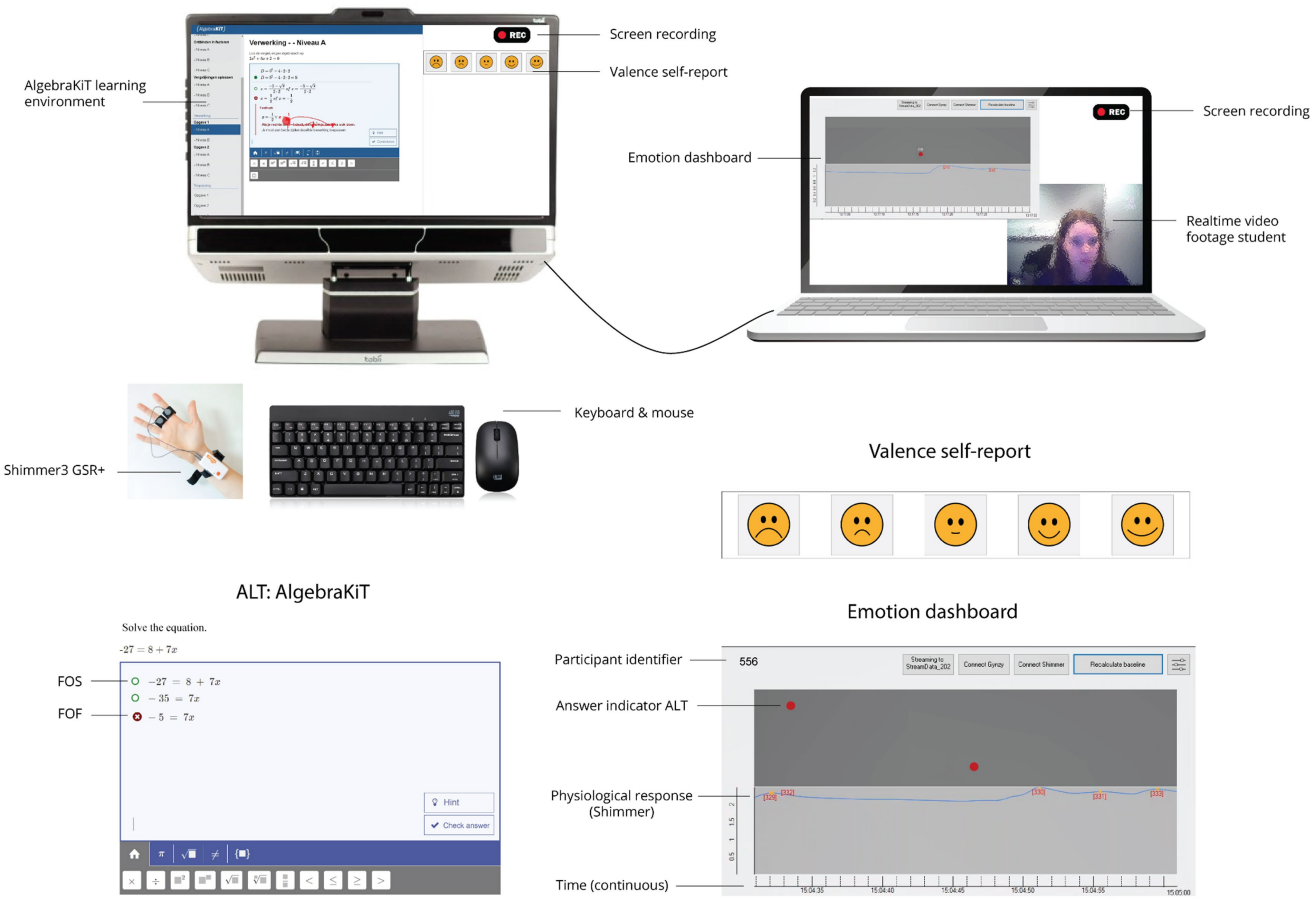
Overall set-up of the experiment.

The ALT used in this study was AlgebraKiT, a web-based software application for practicing mathematical problem-solving (Figure 2). Students entered every calculation they made to arrive at the final solution of the problem into the ALT. The algorithm behind AlgebraKiT analyzed these steps using the calculation principles taught in secondary education. Based on these principles, the ALT determined if a student’s calculation was correct or incorrect and provided students with immediate feedback after every calculation. The task difficulty of tasks was manually adjusted based on students’ pre-test scores.

#### 2.3.2 Feedback types

AlgebraKiT could generate two types of immediate feedback, which were labeled feedback on success (FOS), which was given after a student made a correct calculation, and feedback on failure (FOF), which was given after an incorrect calculation. A green circle indicated a correct calculation and a red circle with a white cross indicated an incorrect calculation (Figure 2). In some cases (105 times), the notification that a calculation was incorrect was followed by an explanation.

#### 2.3.3 Learning objective

The learning objective in this study was the quadratic formula $\left(x=\frac{-b\pm \sqrt{b^{2}-4ac}}{2a}\right)\text{.}$ Students were asked to solve three practice problems with this formula, which required multiple calculation steps. The first problem had students solve a given quadratic equation. In the second problem, students needed to calculate the intersection of two equations. The third problem was of an applied nature in that the formula was embedded in a cover story. Every problem had three difficulty levels to facilitate variation in success and failure in all students:

Easy: e.g. Given are two equations $f\;\left(x\right)$ and $g\;\left(x\right)$ . Solve the equation

$f\;\left(x\right)=g\;\left(x\right)\text{.}$ $f\;\left(x\right)=2-x^{2}-2x$ and g $\left(x\right)=5x-4$

Intermediate: e.g. Given are two equations $f\;\left(x\right)$ and $g\;\left(x\right)$ . Solve the equation

$f\;\left(x\right)=g\;\left(x\right)\text{.}$ $f\;\left(x\right)=8-x\;\left(3-2x\right)$ and g $\left(x\right)=5x^{2}+2+4x\text{.}$

Hard: e.g. Given are two equations $f\;\left(x\right)$ and $g\;\left(x\right)$ . Solve the equation

$f\;\left(x\right)=g\;\left(x\right)\text{.}$ $f\;\left(x\right)=\frac{3}{2}x^{2}+\frac{1}{2}\;x-7$ and g $\left(x\right)=-x\left(\frac{1}{4}x+2\right)-5$

Students were assigned to one of these difficulty levels based on their pre-test score (see section 2.4.3. for more information on the pre-test).

#### 2.3.4 Instruction

Students were taught the quadratic formula via an instruction video taken from a public YouTube account (Jawiskunde, 2017). This 6-min video explained all components of the quadratic formula and demonstrated in two examples how it could be applied in solving math problems.

#### 2.3.5 Nature video

Students watched a 5-min video of different landscape views accompanied by relaxing music (Cat Trumpet, 2019). This video served to establish a baseline for the EDA measurement, which was used to measure students’ physiological arousal.

#### 2.3.6 Emotion dashboard

To ensure the synchronization of the data streams, an emotion dashboard was developed (Figure 2). On this dashboard, the researcher could see a student’s answers to every calculation step and physiological responses on a continuous timeline. The on-screen position of the answer indicator (red circle) indicated whether a calculation was correct (at the top of the dark gray part of the screen) or incorrect (at the bottom of the dark gray part of the screen).

### 2.4 Measurements

#### 2.4.1 Logfile data of the ALT

The ALT stored the following information for each participant: student identifier, exercise identifier, timestamp in milliseconds, and correctness of a calculation.

#### 2.4.2 Background characteristics

The following background characteristics of the participants were orally collected by the researcher: gender, age, field of study, year of study, and level of math in high school.

#### 2.4.3 Pre-test

The pre-test measured students’ prerequisite math knowledge for the quadratic formula. The test consisted of three items about removing brackets (e.g., remove the brackets: $\left(x-12\right)\;\left(x+6\right)$ ) and three items on factorizing (e.g., $7x^{2}+63x+140$ ), increasing in difficulty from easy to hard. One point was awarded for each correct item, so total pre-test scores could range from 0 to 6. Based on students’ pre-test scores, they were assigned to either easy, intermediate or hard practice problems in the main part of the session (see section 2.3.3. for more information on the learning objective). When a student had a score of 2 or less, they were assigned easy problems. A score of 3 or 4 resulted in intermediate problems, and a score of 5 or more in hard practice problems.

#### 2.4.4 Measures of emotional responses

##### 2.4.4.1 Physiological responses

Electrodermal activity (EDA), also called skin conductance, was used to measure physiological arousal throughout the learning session. EDA captures the variation of electrical characteristics of the skin due to sweat gland activity (Dawson et al., 2016). This study used the Shimmer3 GSR+; a wearable device fixed on a wristband with two electrodes placed on the middle phalanges of the index and middle finger of the participant’s non-dominant hand. The researcher verified the correct placement of the wristband at the beginning of each session (participants had to place the electrodes themselves due to COVID-19 regulations). EDA data was recorded using a sampling rate of 51.2 hertz (51.2 raw values measured in micro Siemens (μS) per second).

##### 2.4.4.2 Experiential responses

Self-reports of valence were used to indicate experiential responses. A 5-point scale was used, ranging from very negative to very positive. Based on the Smiley-o-meter (Read, 2008), all five scale values were visualized by an emoticon. The researcher prompted students to indicate how they were feeling after every feedback event. The self-report tool was visible on a split screen next to the AlgebraKiT screen (Figure 2).

##### 2.4.4.3 Behavioral responses

Observations of students’ facial expressions were used to provide insight into the emotion type (Baker et al., 2010). The Baker Rodrigo Ocumpaugh Monitoring Protocol (Ocumpaugh et al., 2015) was used to classify students’ facial expressions as either anxiety, boredom, confusion, disappointment, engaged concentration, enjoyment, frustration, relief or surprise (Pekrun, 2006; D’Mello, 2013). Two observers indicated the facial expression they observed instantly after every feedback event using recordings of the participants’ faces. Before, observers were trained using videos of students working on individual practice problems. After the training phase, the agreement between the observers was 78.5%. The observers discussed their disagreement and adjusted the coding scheme when they agreed on distinct features in the face. Formal interrater reliability was calculated with Cohen’s kappashere was substantial agreement between the two observers, κ = 0.614, *p* < 0.001 (Landis and Koch, 1977; Hallgren, 2012).

### 2.5 Coding of the dependent variables

#### 2.5.1 Coding of physiological responses

**Figure 3:**
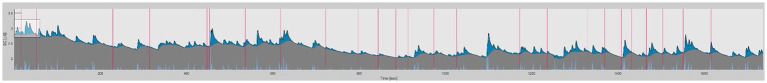
Ledalab screenshot of one participant during the whole learning session (www.ledalab.de; Benedek & Kaernbach, 2010). Tonic component (grey), phasic component (blue), and feedback events (red line).

**Figure 4:**
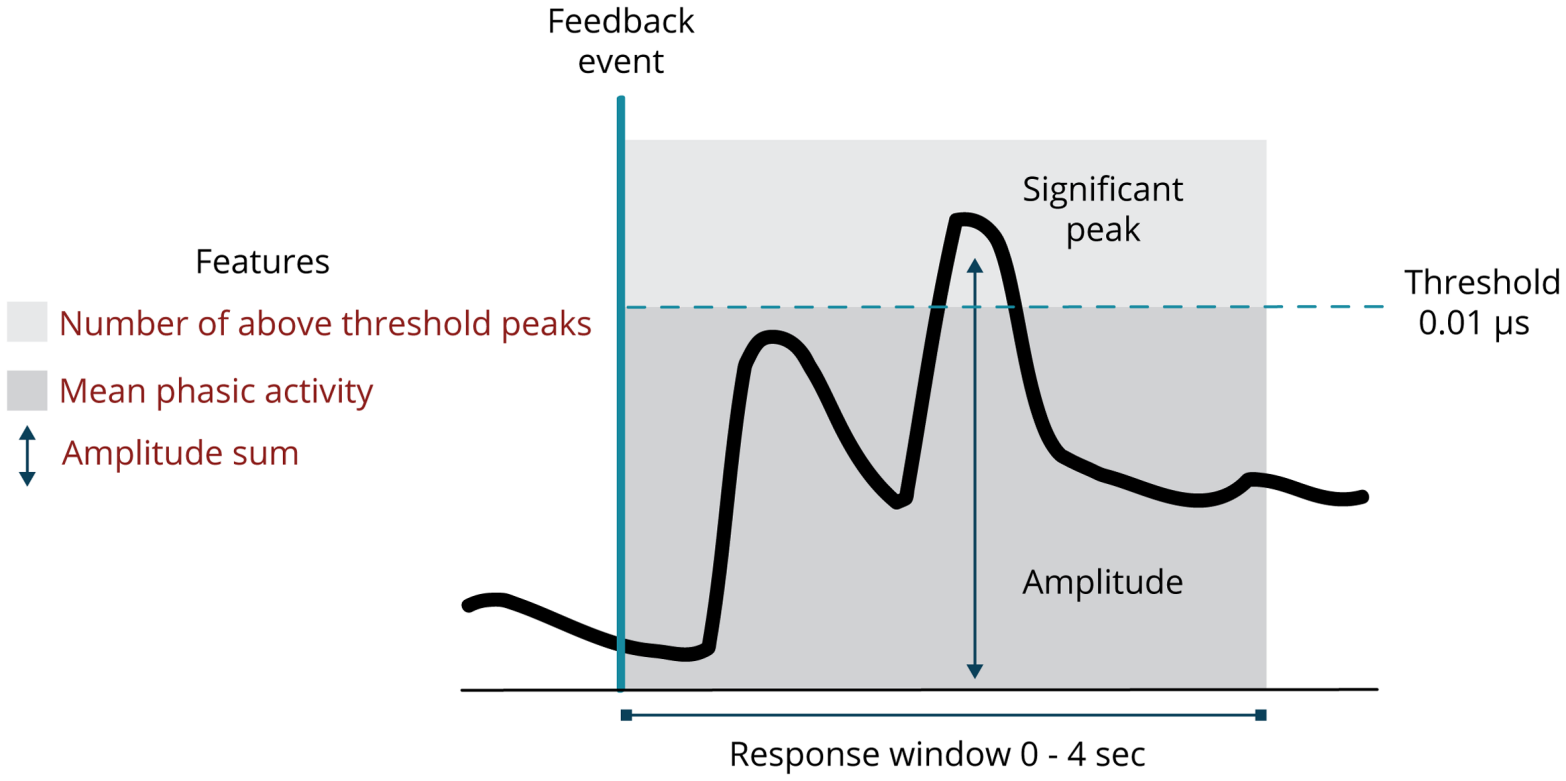
EDA features used in this study.

EDA data were analyzed using the MATLAB Ledalab toolbox (Benedek and Kaernbach, 2010). Movement artifacts were visually identified and manually deleted. No filtering and down-sampling was applied. Feature extraction was obtained via Continuous Deconvolution Analysis (CDA). This analysis divides the EDA signal into a tonic and a phasic component. The tonic component is a slowly varying signal that generates a moving baseline per individual (relatively stable within a few seconds). The phasic component refers to the fast-moving signal, representing faster-changing elements in the EDA signal, i.e., peaks (Braithwaite et al., 2015; Dawson et al., 2016). Data of one participant is shown in Figure 3. Event-related responses after immediate feedback were extracted by this analysis using a response window of plus 4 s, resulting in stimulus-specific features. This response window was chosen because of recommendations in previous research on fast stimuli and EDA latency of 1 to 3 s (Benedek and Kaernbach, 2010; Dawson et al., 2016; Horvers et al., 2021). Three features were extracted from the EDA signal (see Figure 4), all measured within the response window (wrw) based on previous research (Horvers et al., 2021). These features were *the number of above-threshold peaks*, *the amplitude sum of above-threshold peaks*, and *the mean phasic activity.* An increase in EDA could be classified as a significant peak when it is above a certain threshold; 0.01 μs was used as threshold based on previous research (Horvers et al., 2021).

#### 2.5.2 Coding of experiential responses

The five valence options were strong positive, positive, neutral, negative and strong negative. These options were coded according to the 5-point scale: 1 for very negative, 2 for negative, 3 for neutral, 4 for positive, and 5 for very positive. In line with the categorization of emotion types into positive and negative emotion types and neutral facial expressions (see section 2.5.3), the valence options were also merged into three categories, with strong positive and positive in the positive category, strong negative and negative in the negative category and neutral in the neutral category.

#### 2.5.3 Coding of behavioral responses

The observed facial expressions were categorized into negative and positive emotion types and neutral facial expressions. Based on literature, enjoyment (Pekrun and Stephens, 2010; D’Mello, 2013) and relief (Pekrun and Stephens, 2010) were placed in the positive emotion category. Boredom, frustration (Pekrun and Stephens, 2010; D’Mello, 2013), anxiety, disappointment (Pekrun and Stephens, 2010), and confusion (D’Mello, 2013) were placed in the negative emotion category. Engaged concentration was indicated as neutral. Surprise can have negative as well as positive valence (Noordewier and Breugelmans, 2013), due to this ambiguity this emotion was excluded from analyses. After categorization, there was still substantial agreement between the two observers overall (κ = 0.704, *p* < 0.001), and separately for negative emotion types (κ = 0.792, *p* < 0.001) and neutral facial expressions (κ = 0.741, *p* < 0.001). Positive emotion types had a moderate agreement (κ = 0.429, *p* < 0.001) (Landis and Koch, 1977; Hallgren, 2012). As a next step, the positive and negative emotion types were divided in activating and deactivating emotions (Pekrun et al., 2007; D’Mello et al., 2014). Enjoyment is categorized as positive activating and relief as positive deactivating. Frustration, anxiety and confusion are categorized as negative activating and boredom and disappointment are negative deactivating (Pekrun et al., 2007; D’Mello et al., 2014).

### 2.6 Data analysis

For the analysis of physiological responses, the number of above-threshold peaks, the amplitude sum of above-threshold peaks, and the mean phasic activity were used. A one-sample t-test analyzed whether immediate feedback triggered an above-threshold peak in EDA at above-chance level. Repeated measures MANOVA was used to examine differences in students’ physiological responses between FOF and FOS. Dependent variables were the proportion of above-threshold peaks, the amplitude sum and the mean phasic activity. For the analysis of experiential responses, the proportions of positive valence and negative valence were used, averaged per participant. To examine students’ experiential responses, repeated measures ANOVAs with Greenhouse–Geisser correction were run separately for FOF and FOS with the proportions of negative and positive valence as dependent variables. For the analysis of behavioral responses, the proportions of positive emotion types and negative emotion types were used, averaged per participant. A similar approach was used to examine within-subject differences in behavioral responses, with the proportions of negative and positive emotion types as dependent measures. To investigate the difference in deactivating and activating emotion types, repeated measures ANOVAs were used.

## 3 Results

Students received immediate feedback 990 times in total. The average number of feedback events per student was 30.94 (*SD* = 11.99). Students received feedback on failure (FOF; *M* = 15.03, *SD* = 9.09) about as often as feedback on success (FOS; *M* = 15.56, *SD* = 7.83).

### 3.1 Physiological responses

Feedback yielded 1.17 above-threshold peaks in EDA on average (maximum of 5 above-threshold peaks). To investigate whether feedback triggered an above-threshold peak at an above-chance level, a one-sample *t*-test was run. This test indicated that the proportion of above-threshold peaks was significantly higher than chance level (0.50), *t*(31) = 5.965, *p* < 0.001.

Table 1 shows the descriptives of physiological responses for the three features that are extracted from the EDA signal (proportion of above-threshold peaks, amplitude sum and mean phasic activity). The columns show the grand means of these three variables and the means for FOF and FOS. The mean proportion of above-threshold peaks was slightly lower for FOF (0.73) than for FOS (0.77). The average amplitude sum of the above-threshold peaks was 0.24, and slightly higher amplitudes were observed for FOF (0.28) than for FOS (0.23). The same goes for the mean phasic activity. Repeated measures MANOVA showed that these minor differences were not statistically significant, *F*(3, 30) = 1.682, *p* = 0.193, partial η^2^ = 0.153.

**Table 1:** Descriptives of students’ physiological responses to feedback.

	Total		FOF		FOS	
	*M*	*SD*	*M*	*SD*	*M*	*SD*
Proportion of above-threshold peaks	0.75	0.24	0.73	0.27	0.77	0.26
Amplitude sum	0.24	0.26	0.28	0.34	0.23	0.26
Mean phasic activity	0.34	0.35	0.39	0.45	0.32	0.32

FOF, feedback on failure; FOS, feedback on success. *N* = 32.

### 3.2 Experiential responses

Students reported the valence of their emotions 26.13 times on average during the learning session (*SD* = 11.05). Valence was indicated after 84.4% of the feedback events, and no indication of valence was given after 15.6% of the feedback events. For FOF, the valence was indicated 11.84 times on average (*SD* = 8.03), and for FOS slightly more often (*M* = 14.28, *SD* = 7.04).

On average, students reported more positive valence (0.47) than negative valence (0.30), although the occurrence rates differed considerably based on the type of feedback (Table 2). Repeated measures ANOVA with Greenhouse–Geisser correction showed a significant difference between the proportion negative and positive valence after FOF, *F*(1, 31) = 84.274, *p* < 0.001, partial η^2^ = 0.731. This result indicates that students predominantly expressed negative valence after FOF. A reverse pattern was found for FOS, where the proportion of positive valence exceeded the proportion of negative valence. Repeated measures ANOVA with Greenhouse–Geisser correction indicated that this difference was statistically significant, *F*(1, 31) = 252.996, *p* < 0.001, partial η^2^ = 0.891.

**Table 2:** Descriptives of students’ experiential responses to feedback.

	Total		FOF		FOS	
	*M*	*SD*	*M*	*SD*	*M*	*SD*
Proportion positive valence	0.47	0.21	0.06	0.10	0.77	0.24
Proportion negative valence	0.30	0.21	0.66	0.30	0.02	0.05
Proportion neutral valence	0.23	0.16	0.28	0.26	0.21	0.24

FOF, feedback on failure; FOS, feedback on success. *N* = 32.

### 3.3 Behavioral responses

Students’ emotion type was observed 29.00 times on average (*SD* = 11.97). There were slightly higher frequencies for FOS (*M* = 15.21, *SD* = 7.77) than for FOF (*M* = 12.65, *SD* = 7.88). Emotion types were recorded after 92.4% of the feedback events; the remaining 7.6% of the events had missing observations.

Engaged concentration was the prevailing facial expression (0.71) (Table 3). For FOF, a trend was visible with higher proportions of negative emotion types than positive emotion types on average. Repeated measures ANOVA with Greenhouse–Geisser correction showed a significant difference between negative and positive emotion types for FOF, *F*(1, 31) = 48.044, *p* < 0.001, partial η^2^ = 0.608. This indicates that students showed significantly more negative emotion types after FOF than positive emotion types. Students showed significantly more negative activating than negative deactivating emotion types after FOF, *F*(1, 31) = 38.277, *p* < 0.001, partial η^2^ = 0.533. Regarding FOS, the proportions of positive emotion types exceeded the negative emotion types on average. Repeated measures ANOVA with Greenhouse–Geisser correction produced a significant difference between negative and positive emotion types, *F*(1, 31) = 10.888, *p* = 0.002, partial η^2^ = 0.260. This indicates that students showed more positive emotion types after FOS than negative emotion types. Students showed significantly more positive activating than positive deactivating emotion types after FOS, *F*(1, 31) = 13.366, *p* < 0.001, partial η2 = 0.301.

**Table 3:** Descriptives of students’ behavioral responses to feedback.

	Total		FOF		FOS	
	*M*	*SD*	*M*	*SD*	*M*	*SD*
*Proportion positive emotion types*	*0.10*	*0.11*	*0.03*	*0.06*	*0.15*	*0.18*
Enjoyment	0.09	0.10	0.03	0.06	0.13	0.17
Relief	0.01	0.02	0.00	0.00	0.02	0.04
*Proportion negative emotion types*	*0.19*	*0.15*	*0.39*	*0.29*	*0.03*	*0.08*
Anxiety	0.01	0.00	0.03	0.12	0.00	0.00
Boredom	0.00	0.01	0.00	0.00	0.00	0.01
Confusion	0.10	0.11	0.18	0.19	0.03	0.08
Disappointment	0.03	0.04	0.06	0.12	0.00	0.00
Frustration	0.06	0.08	0.11	0.14	0.00	0.00
*Proportion neutral emotion type*	*0.71*	*0.19*	*0.55*	*0.30*	*0.81*	*0.18*
Engaged concentration	0.71	0.19	0.55	0.30	0.81	0.18

FOF, feedback on failure; FOS, feedback on success. *N* = 32.

## 4 Discussion

The main purpose of this study was to explore university students’ emotional responses to immediate feedback provided by an ALT. The first goal of this study was to investigate students’ physiological responses by analyzing to what extent immediate feedback triggers physiological arousal as measured by peaks in EDA. The results indicate that the proportion of above-threshold peaks after feedback exceeded chance level. Physiological responses were not stronger after FOF than FOS. The second goal of this study was to examine students’ experiential and behavioral responses to different types of immediate feedback. The results show that students’ experiential responses entailed mostly positive valence after FOS and mostly negative valence after FOF. Regarding behavioral responses, FOF elicited significantly more negative than positive emotion types and significantly more positive than negative emotion types were elicited by FOS. FOF elicited significantly more negative activating than deactivating emotion types, and significantly more positive activating than deactivating emotion types were elicited by FOS.

In line with hypothesis 1, there were more peaks in EDA after immediate feedback than would be expected based on chance. This result indicates that feedback likely elicits an increase in physiological arousal, or at least that variations will occur after feedback, as was found in previous research (Aghaei Pour et al., 2010; Malmberg et al., 2019a). Hypothesis 2 predicted that FOF would elicit stronger physiological responses than FOS, as previous research indicated particularly strong emotions after failure (Rowe et al., 2014; Hill et al., 2021). This hypothesis was not supported by the results, as the three physiological arousal indicators showed an inconsistent pattern. Although there are slightly different values for the amplitude sum of above-threshold peaks and the mean phasic activity, FOF indeed yielded slightly higher values than FOS, but not significantly. In contrast, FOS produced slightly more above-threshold peaks in EDA than FOF, but also not significantly. These divergent results are potentially due to the analysis method, which could have overestimated the number of above-threshold peaks (Thammasan et al., 2020). A possible solution is to use sparse recovery methods or accelerometer data (Kelsey et al., 2018; Thammasan et al., 2020). This can be a result of low power, as a post-hoc power analysis showed that the sample size was too small (power: 21.9%). Moreover, the frequency of peaks could also have been overestimated because of the chosen threshold. In previous research, a threshold of 0.05 μs instead of 0.01 μs is often used as well (Pijeira Díaz, 2019; Malmberg et al., 2019b). However, these studies mostly used older devices for which the standard is 0.05 μs (Horvers et al., 2021). To conclude, electrodermal activity can be a valuable objective indicator of emotional responses after immediate feedback but should at least be accompanied by one other data stream in order to fully understand students’ emotional responses. The valence scale used in this study also gained insights into the strength of the emotional response, future research could combine this with the arousal data to gain insights into the strength of emotions.

Previous research on the relationship between feedback and emotions is typically mixed, but most studies found that FOF elicits negative emotions and FOS leads to positive emotions (D’Mello et al., 2010; Lipnevich et al., 2021). The present study replicates these findings and, hence, substantiates hypothesis 3. An interesting result is that students indicated more positive valence overall, even though there were comparable numbers of FOF and FOS events in this study. This is an argument for using multimodal data streams to pinpoint what actually happens after FOF and FOS. Similar results were obtained for students’ behavioral responses to immediate feedback. As predicted by hypothesis 4, significantly more negative than positive emotion types occurred in observations following FOF and more positive than negative emotion types in observations following FOS. After both FOF and FOF, activating emotion types occurred significantly more than deactivating emotion types. These results extend existing emotion and feedback theories by showing that emotional responses to immediate and delayed feedback are comparable. Moreover, this study shows that both valence as measured by self-report and observations of emotion types via facial expressions can be used as additional data streams to understand emotional responses.

This study makes a significant scientific contribution to the field of emotions during learning and specifically emotional responses to immediate feedback. The unique contribution of this study is, firstly, its focus on immediate feedback on every calculation students enter into the ALT, as most previous research focused on delayed feedback or feedback after each task (Jarrell et al., 2017; Hill et al., 2021). Secondly, contrary to prior research, which mainly relied on retrospective measures of emotions (Jarrell et al., 2017; Hill et al., 2021), this study measured emotions during the learning process by prompting students to indicate valence and observing their facial expressions after every feedback event and continuously measuring physiological arousal. Lastly, this study uses a multimodal approach instead of a unimodal approach. Most research on the relationship between feedback and emotion used a single data stream (Lim et al., 2020; Hill et al., 2021). This unimodal approach has some constraints, such as the possibility for participants to control their self-reported answers (Pekrun, 2020). The multimodal approach used in this study overcomes these constraints by using continuous measures of arousal (physiological responses), valence self-reports (experiential responses) and observations of emotion type via facial expressions (behavioral responses) to capture emotional responses. This combination of measures is in line with recommendations to view emotions as multi-componential in nature (Harley et al., 2015; Li et al., 2021). This study is one of the first to show how multimodal measurement of emotional responses to immediate feedback in the context of adaptive learning technologies can be performed. However, future research should combine the multimodal data streams in their analyses, to gain even more insight in the multi-componential nature of emotion.

A limitation of this study is that data were collected in the research laboratory. Previous research has substantiated the importance of investigating emotional responses in authentic settings because students may show different responses under controlled circumstances (D’Mello, 2013). Our results may, therefore, not generalize to students’ regular classes at the university because not all universities use ALTs in their daily classes and teaching yet, but some examples exist (Gillebaart and Bellinga, 2018). However, 60 to 70% of the pupils in Dutch primary schools use ALTs on a daily basis (Van Wetering et al., 2020; Horvers et al., 2021). Contrary to the ALT used in this study, these primary education ALTs mostly automatically adjust the difficulty of tasks to the ability level of students (Klinkenberg et al., 2011; Van Wetering et al., 2020). Therefore, we recommend replicating the present study with a younger group of learners.

Another potential direction for further research would be to compare physiological responses to feedback to the occurrence and magnitude of other peaks in EDA during the learning process. This could show which variations there are after feedback compared to other moments in learning. An additional suggestion for future research would be to extend all measurements of emotion beyond feedback events, not only physiological arousal. Experiential and behavioral responses could be measured whenever a peak in arousal occurs to investigate learning processes in a fine-grained manner. The multimodal data streams that are used provide insights into a detailed level of emotional responses by using a continuous measure of physiological arousal and self-reports and observations after each calculation step students take. Future research could compare physiological, experiential and behavioral responses to feedback to these responses at other critical moments in learning, such as calculating an answer or receiving a new problem (Fritz et al., 2014).

Adaptive learning technologies currently only use students’ cognitive achievements to base the difficulty level of problems and immediate feedback on (Klinkenberg et al., 2011). However, as emotions can both hamper and improve learning, there is an opportunity for ALTs to take students’ emotions into account, thereby moving from “cold” to “warm” technologies (Götz and Hall, 2013; Loderer et al., 2020). The results of this study indicate that students mostly show negative emotional responses to FOF and positive emotional responses to FOS, but as these are averages, these responses do not always occur. Sometimes students can react negatively to FOS as well, as also indicated by previous research, for instance, by eliciting boredom when a task is too easy (Pekrun, 2006; Inventado et al., 2011). Addressing these differences in emotional responses to feedback is an important first step to move from cold to warm technologies. The effects of these emotional responses to feedback on learning outcomes should be addressed as well in future research. Moreover, individual differences between students should be taken into account because some students prefer easy tasks to avoid negative emotions and others experience less emotions when they fail than others when challenged by difficult tasks (Baker et al., 2010). A next step for future research is to investigate these individual differences in emotions even more by looking at emotional responses to different difficulty levels of tasks, to ultimately arrive at warm technologies that can accommodate these individual differences.

## 5 Conclusion

This study is an important first step to understanding students’ emotional responses to immediate feedback and consequently moving from cold to warm technologies. Results show that immediate feedback often elicits peaks in EDA and thus high physiological arousal — which is generally taken as a sign that an emotion is triggered — with no differences in strength between feedback on failure or feedback on success. This result indicates that multiple data streams are needed to capture emotional responses. Both experiential responses measured by self-reported valence and behavioral responses measured by observations of emotion types can be used as an additional data source. Feedback on failure elicits predominantly negative emotions, while feedback on success elicits mostly positive emotions. Both feedback on failure and success elicited mostly activating emotions. To conclude, these results imply that emotional responses to immediate feedback can be validly assessed from multimodal data streams, which aligns with the theoretical notion that emotions are multi-componential in nature. These insights provide a good starting point for going from cold to warm technologies that take students’ emotions into account.

## Data availability statement

The raw data supporting the conclusions of this article will be made available by the authors, without undue reservation.

## Ethics statement

The studies involving humans were approved by the Ethics Committee Social Sciences (ECSS) of the Radboud University. The studies were conducted in accordance with the local legislation and institutional requirements. The participants provided their written informed consent to participate in this study.

## Author contributions

AH: Conceptualization, Data curation, Formal analysis, Methodology, Visualization, Writing – original draft, Writing – review & editing. IM: Conceptualization, Methodology, Writing – review & editing. HV: Data curation, Writing – review & editing. TB: Conceptualization, Methodology, Writing – review & editing. AL: Conceptualization, Methodology, Writing – review & editing.
